# Trimming the fat: a brief review of lipids at the host-pathogen interface

**DOI:** 10.1128/iai.00506-24

**Published:** 2025-06-13

**Authors:** Filiz T. Korkmaz

**Affiliations:** 1 Department of Microbiology and Immunology, University of Iowa Carver College of Medicine 12243 , Iowa City, Iowa, USA; University of Pittsburgh, Pittsburgh, Pennsylvania, USA

**Keywords:** host-pathogen interactions, immune mechanisms, lipid signaling, infectious disease

## Abstract

Microbial-derived lipids and the host receptors that bind them are collectively critical for immune regulation on the host side and for a multitude of biological functions on the microbial side, including membrane structure, energy generation, resistance to stress, and, importantly, virulence. Bacteria, viruses, fungi, and eukaryotic microorganisms comprise common and unique lipid species that can be modified to avoid immune detection and aid in antimicrobial resistance. Moreover, the host receptors that interact with lipids are equally diverse in their structure and function, driving both beneficial and pathogenic responses depending on the location, strength, and duration of signaling. The following review will discuss all the aforementioned aspects of lipids at the host-pathogen interface, which should be expanded upon in future studies to develop novel therapeutics that consider lipids as distinct immune modulators.

## INTRODUCTION

Lipids represent a complex and varied group of molecules used for energy storage and consumption, signaling molecules, and membrane structure. Both host and microbial lipids are structurally and functionally diverse, ranging from phospholipids to isoprenoids, sterols, glycolipids, and fatty acids. However, microbial membranes and cell walls comprise unique lipid species, allowing specific interactions with host receptors (Table 1). Some examples are lipopolysaccharides in gram-negative bacteria and mycolic acids in *Mycobacterium* spp. of bacteria (1). Moreover, viral envelopes that protect the genetic material of viruses are surrounded by a phospholipid bilayer while also exploiting host cell lipids for entry, replication, and egress (2). While not the focus of this review, host-derived lipids play a critical role during the response to infection through the organization of lipid rafts, fatty acid synthesis, and oxidation, and through the generation of signaling molecules (sphingosine 1-phosphate, eicosanoids, and maresin) (3–5), among others.

**TABLE 1:** Host receptors for microbial lipids and lipoproteins

Receptor	Receptor class	Lipid ligand(s)
TLR2	Toll-like Receptor	Lipotechoic Acid
TLR4	Toll-like Receptor	Lipopolysaccharide, Lipid A, Cardiolipin
Mincle	C-type Lectin	trehalose-6,6′-dimycolate, glyceroglycolipid, mannosyl fatty acids, LTA anchor molecules (mono- and diglucosyldiacylglycerol), glucosyl-diacylglycerol, cholesteryl acyl α-glucosides
DC-SIGN	C-type Lectin	Lipopolysaccharide, Lipoarabinomannan
Dectin-2	C-type Lectin	mannosylated LPS
DCAR	C-type Lectin	acylated phosphatidyl-*myo*-inositol mannosides
CD206	C-type Lectin	Lipoarabinomannan
Marco	Class A Scavenger Receptor	Lipoarabinomannans, lipopeptides
SR-A	Class A Scavenger Receptor	Acetylated LDL
SR-B1	Class B Scavenger Receptor	SARS-CoV2 via HDL
CD36	Class B Scavenger Receptor	Lipopolysaccharide, Lipomannan, Lipoarabinomannan, Lipotechoic Acid
SR-PSOX	Class B Scavenger Receptor	Lipopolysaccharide
LOX-1	Class E Scavenger Receptor	Unknown
CD1d	CD1	dideoxymycobactin, glucose monomycolate, mannosyl-1B-phosphomycoketide, phosphatidylinositol mannoside-4, cardiolipin
Caspase-4	Cysteine Protease	lipid A
GBP1	N/A	O-antigen
NLRP7	Nod-like Receptor	Acylated bacterial lipopeptides
NLRP3	Nod-like Receptor	Ornithine lipid

With few exceptions (e.g., TLR4/lipopolysaccharide [LPS]), host receptor-lipid interactions are less well-characterized mechanisms of generating immunity to infectious disease. However, many additional receptors bind to lipids, leading to an array of biological responses that either help or hinder response to infection. Here, we will review host receptors that utilize lipid molecules as their cognate ligands (Table 1), lipid species that are expressed by pathogenic microorganisms (Table 2), and the consequences of these interactions on response to infection. We will also address the multiple gaps in knowledge surrounding this critical area of infectious disease-related research and how future studies could lead to novel therapeutic options.

**TABLE 2:** Microbial lipids and lipoproteins with known host interactions

Lipid	Receptor	Microorganism	Known outcomes
Sterylglycoside	Unknown	Various fungi	Promotes CD4 +T cell activation
Glycosylceramide	Mincle	Various fungi	Enables growth of *Cryptococcus*
α-Galactosylceramide	CD1d	*Bacteroides* spp	Immune homeostasis, protection against vaginal candidiasis
Lipomannan	TLR2, TLR4, MR, CRs, CD36	*Mycobacterium spp*	Promotes macrophage apoptosis, TNF inhibition
Lipoarabinomannan	DC-SIGN, CD206, CRs, Dectin-2, Marco, CD36	*Mycobacterium spp*	Anti-apoptotic, cytokine activation & inhibition, phagocytosis or promotion of bacterial replication (receptor-dependent)
Glycopeptidolipids	Mannose receptor	*Mycobacterium spp*	Reduces drug permeability, delay in phagolysosome maturation
Phthiocerol dimycocerosate	CR3	*Mycobacterium spp*	Inhibits LC3-mediated phagocytosis, antibiotic resistance
Acetylated LDL	SR-A	*Neisseria meningitidis*	Pro-inflammatory cytokine activation
Lipotechoic Acid	TLR2/6, CD14, SR-A, CD36	Gram-positive bacteria	Bacterial clearance, activation or prevention of inflammation
Lys-Glc-DAG	Unknown	*Streptococcus agalactiae*	Facilitates crossing of BBB
High density lipoprotein	SR-B1	SARS-CoV2	Promotes viral uptake
Cardiolipin	TLR4/MD2	Ubiquitously expressed	NLRP3 inflammasome activation, TLR4 inhibition
Lipopolysaccharide; Lipid A	TLR4/MD2, SR-PSOX, CD36	Gram-negative bacteria	Pro-inflammatory activation, TNF-α production
Ornithine lipid	NLRP3; TLR4	Gram-negative bacteria	TLR4/NLRP3 activation in phosphate-limited environments; Stress resistance
PAL	Unknown	*Burkholderia cenocepacia*	Host cell attachment & activation of inflammation
Ergosterol	Unknown	*Candida albicans*	Promotes biofilm formation
*N*-palmitoyl serinol	GPR119	Commensals	Immune & metabolic homeostasis
*N*-3-hydroxypalmitoyl ornithine	S1PR4	Commensals	Immune & metabolic homeostasis
*N*-myristoyl alanine	G2A	Commensals	Immune & metabolic homeostasis

## LIPID-RESPONSIVE RECEPTORS

Toll-like receptors, first described in 1994, are germline-encoded pattern recognition receptors, expressed either on the cell surface or on endosomes, that recognize conserved molecular patterns on microbe and host alike (6). TLRs are expressed on the majority of immune cells (DCs, macrophages, neutrophils, and B-cells), along with sentinel structural cells, such as epithelial cells, endothelial cells, and fibroblasts (7, 8). Moreover, they are the most widely recognized and well-characterized host receptors that recognize bacterial lipids and lipoproteins, among other ligands. While TLRs and their binding partners have been reviewed in detail elsewhere (9), their interactions with their ligands have clear roles in promoting inflammation by signaling through TIR domains, leading to the recruitment of adaptor proteins (e.g., MyD88, MAL, and TRIF) and activation of transcription factors such as NFkB and IRF3 (10). These interactions provide critical defense against pathogens, evidenced by patients with TLR pathway deficiencies who are more susceptible to infection with herpes simplex virus-1 (11), *Streptococcus pneumoniae*, *Pseudomonas aeruginosa,* and Staphylococci (12, 13), while overactivation of TLRs may lead to autoimmunity (6, 14).

However, in the context of responses to lipid ligands, TLR2 primarily recognizes di- and tri-acylated lipoproteins, expressed by gram-positive and gram-negative bacteria, respectively. *Staphylococcus aureus* has been shown to induce IL-8 production in a TLR2-dependent manner in intestinal epithelial cells through the expression of lipoproteins. This interaction may explain how this pathogen can induce intestinal inflammation (15). In fact, intestinal epithelial cells express a wide array of TLRs, though expression is regulated both spatially and by cell type, where they interact with commensal and pathogenic bacteria (16). Moreover, TLR2 activation also results from an *S. aureus-*derived tri-acylated lipoprotein, resulting in MyD88-dependent activation of IL-6 and TNF-α from thioglycolate-elicited peritoneal macrophages (17). Staphylococcal lipoproteins also induce the production of nitric oxide in primary mouse macrophages (18). Meanwhile, TLR4 is the canonical receptor for the glycolipid, lipopolysaccharide, expressed by gram-negative bacteria. This interaction, reviewed in great detail elsewhere (19, 20), activates potent and potentially dangerous inflammatory signaling through extracellular and intracellular mechanisms and its various adaptor proteins, including MyD88 and TRIF. In addition to TLRs, C-type lectin receptors, NOD-like receptors, scavenger receptors, and CD1 molecules bind to host and microbe-derived lipids.

C-type lectin receptors (CLRs) are a class of receptors that contain a C-type lectin domain, defined by their Ca^2+^-dependent carbohydrate binding, including lectin, as its name suggests (21). While most data on CLRs are focused on macrophages due to their role as professional phagocytes that exhibit high levels of CLR expression, they are also expressed on dendritic cells, monocytes, and neutrophils. Moreover, certain CLRs may be expressed on non-immune cells, such as endothelial cells and smooth muscle cells, as is the case for lectin-like oxidized low-density lipoprotein receptor-1 (LOX-1) (22, 23). CLRs provide protection against a variety of pathogenic microorganisms, including *Mycobacterium tuberculosis, Candida albicans,* and *Aspergillus fumigatus,* among others (24). Interestingly, the expression of the C-type lectin receptor, CLEC5A, promotes the progression of dengue hemorrhagic fever through DAP12 phosphorylation and pro-inflammatory cytokine production (25). Like TLRs, aberrant C-type lectin signaling can also promote allergy and autoimmunity (26).

Macrophage-inducible C-type lectin, or Mincle, is a member of the CLR family with known lipid-binding properties and is primarily expressed by myeloid cells (27). Importantly, trehalose-6,6′-dimycolate), a component of the *M. tuberculosis* cell wall, is recognized by Mincle and mediates anti-bacterial immunity to this nefarious pathogen (28, 29). Mincle associates with FcRγ to activate the Syk-Card9 pathway, promoting the production of cytokines, nitric oxide, and inducing Th1/Th17 immunity (30, 31). *Malassezia,* an opportunistic fungal pathogen of the skin, is also recognized by Mincle in cooperation with Dectin-2. Here, the identified Mincle ligands were glyceroglycolipid and mannosyl fatty acids linked to mannitol, leading to NFAT activation and cytokine production (32). Additionally, Mincle recognizes group A *Streptococcus* (GAS) through a lipotechoic acid anchor molecule, monoglucosyldiacylglycerol, activating antibacterial immunity through Card9/NFAT (33). Mincle also binds to a second GAS LTA anchor molecule, diglucosyldiacylglycerol. However, this was shown to inhibit Mincle signaling, perhaps as an immune evasion mechanism by GAS (33). Finally, Mincle regulates the response to lung infection by interacting with a glycolipid expressed by *Streptococcus pneumoniae*. Here, the expression of Mincle on hematopoietic cells is protective against lethal pneumococcal infection, at least partially mediated by its interaction with the glycolipid, glucosyl-diacylglycerol, which promotes inflammatory cytokine expression on alveolar macrophages (34).

DC-SIGN, a CLR with a strong affinity for mannose-type N-glycans, also binds to lipopolysaccharides, including those with Lewis epitopes, a form of molecular mimicry used by *Helicobacter pylori*, a gram-negative pathogen that can reside in the stomach, causing ulcers and stomach cancer (35). However, this interaction suppresses the activation of gastric dendritic cells, leading to lower levels of IL-6- and Th1-mediated immunity (36). *H. pylori* also generates secreted metabolites that are derived from cholesterol, specifically, cholesteryl acyl α-glucosides (37, 38), which were shown to bind to Mincle. The interaction between α-CAG and Mincle is pathogenic in the development of gastritis, promoting Th1/Th17 responses with no impact on bacterial clearance (39). DC-SIGN also binds to LPS of other bacterial species, including *Yersinia pseudotuberculosis*, specifically to the core O-antigen. Here, this interaction inhibits the dissemination of bacteria, as bacteria lacking the O-antigen of LPS exhibit higher levels of invasiveness (40). Dectin-2, a CLR with a significant role against fungal pathogens (41), recognizes mannosylated LPS on the nosocomial pathogen *Hafnia alvei,* working in concert with TLR4 to enhance pro-inflammatory cytokine expression (42). Dendritic cell immunoactivating receptor (DCAR, or *Clec4b1*), a CLR that has structural similarity to Mincle, binds to unique phosphoglycolipids known as acylated phosphatidyl-*myo*-inositol mannosides (AcPIMs), which are found on *Mycobacterium* spp*.* (43). Expression of DCAR on monocyte-derived inflammatory cells leads to cytokine expression when stimulated with AcPIM2, leading to Th1 activation and decreased lung CFU during *M. tuberculosis* infection (44).

Scavenger receptors, some of which are also C-type lectin receptors, are a diverse family of receptors that are classified on their structure and bind a tremendous variety of ligands, including microbial and host-derived lipids and lipoproteins (45). They are also widely expressed among cell types, including adipocytes, smooth muscle cells, epithelial cells, macrophages, T and B cells, dendritic cells, and neutrophils, with endothelial cells being a major driver of scavenger receptor research (45–47). Due to their ability to bind modified lipoproteins, specifically oxidized low-density lipoprotein (oxLDL), numerous scavenger receptors (CD36, LOX-1, MARCO, SR-B1, SR-PSOX, and SR-A1) have been shown to contribute to atherosclerosis progression (48, 49). In particular, CD36, a class B scavenger receptor that facilitates responses to a number of DAMPs and PAMPs, promotes the uptake of oxLDL, leading to inflammasome activation, ROS production, and NfκB signaling (50). As such, CD36 has been shown to contribute to a number of chronic inflammatory conditions, such as atherosclerosis, Alzheimer’s disease, and type 2 diabetes (51). In the context of infection, CD36 can partner with TLR2 to bind to lipoteichoic acid to facilitate response to gram-positive pathogens, such as *S. pneumoniae* and *S. aureus,* where CD36 knockout mice are hypersusceptible to *S. aureus* infection (52, 53) and are immunosuppressive in response to pneumococcal infection (54). CD36 also interacts with LPS on gram-negative bacteria and lipomannan and lipoarabinomannan expressed on *Mycobacterium* spp. to trigger phagocytosis, cytokine signaling, and Jnk signaling (55–57).

Marco, a class A scavenger receptor, binds to modified lipoproteins, among other ligands, facilitating phagocytosis of pathogens and tumor cells (45, 58). Polymorphisms in Marco have been correlated with susceptibility to TB infection as it can bind and respond to lipoarabinomannans and lipopeptides, similar to CD36 (59). Scavenger receptors SR-A and Marco both bind to *Neisseria meningitidis*, although their interacting partners are unique. Acetylated LDL from *N. meningitidis* is sufficient to induce cytokine production via SR-A, while Marco activation is independent of acetylated LDL and may rely on Neisserial proteins and TLR dimerization (60). SR-B1 is a receptor for HDL, and recent evidence has shown that it promotes the uptake of SARS-CoV-2 on kidney epithelial cells indirectly through virus-HDL binding (61). LOX-1, a class E scavenger receptor and another prominent activator of cardiovascular injury and anti-inflammatory activity in the lung, also appears to bind gram-positive and gram-negative bacteria, although information on this is limited and it may actually occur through binding with poly(I). Finally, SR-PSOX (CXCL16) promotes TNF-α production in response to LPS and dextran sulfate, though the consequences of this in infection remain unexplored (55).

CD1 molecules are a class of receptors with structural similarity to MHC class I molecules. They predominately bind to endogenous and exogenous glycolipid or lipid antigens and may have evolved as a mechanism to respond to Mycobacterium. As such, they bind to numerous lipid molecules uniquely produced by Mycobacterium, including dideoxymycobactin, glucose monomycolate, mannosyl-1B-phosphomycoketide, and phosphatidylinositol mannoside-4. CD1 molecules survey the mucosal tissue environment, where they contribute to T-cell-mediated immunity and response to infection, but also atopic dermatitis, psoriasis, asthma, and COPD. They are expressed on antigen-presenting cells, including, but not limited to, dendritic cells, macrophages, B cells, and epithelial cells, where they bind and present lipid antigens to natural killer T-cells (NKT) (62). NKT cells are divided into two classes, type I and type II NKT cells, based on their T-cell receptor (TCR) usage, where type I cells express an invariant TCRα-chain and type II cells express varied TCRs, making them more challenging to study (63). While type I NKT cells primarily bind to α-galactosylceramide (α-GalCer), type II NKT cells (or dNKT cells) bind to a larger group of microbial-derived lipids. dNKT cells respond to both microbial and host-derived lipids; however, for the purposes of this review, we will focus on microbe-specific ligands. Hybridoma co-culturing studies showed that dNKT cells bind to *M. tuberculosis* and *Corynebacterium glutamicum-*derived lipid molecules, including diphosphatidylglycerol, or cardiolipin, phosphatidylinositol, and phosphatidylglycerol, all of which stimulated the production of IL-2 when dNKT hybridomas were cultured with CD1d-expressing Raw 264.7 macrophages (64). Similar studies were performed with lipid extracts derived from the pathogen, *Listeria monocytogenes*, which identified *L. monocytogenes-*derived phosphatidylglycerol as a potent activator of dNKT cells through CD1d (65).

Caspase-4, an intracellular cysteine protease of the caspase family and a noncanonical inflammasome that promotes pro-inflammatory cell death is expressed fairly ubiquitously among immune and non-immune cells, including macrophages, neutrophils, fibroblasts, epithelial and endothelial cells (66, 67). It is activated by Lipid A molecules found on the extracellular surface of Gram-negative bacteria as part of the lipopolysaccharide outer membrane. Specifically, it has been shown that human caspase-4, but not the mouse ortholog caspase-11, is activated by under-acylated lipid A molecules from *Francisella novicida* (68). Though not receptor-based, outer membrane vesicles (OMVs) make their way into non-phagocytic cells through clathrin or caveolin-dependent endocytosis or through fusion with lipid rafts (69). In this way, extracellular bacteria can activate intracellular death pathways, such as was shown by the activation of caspase-11 during *E. coli* infection, where caspase activation depended upon the delivery of LPS with OMVs (70). As described in more detail below, OMVs carry a variety of lipid species that impact immunity to infection.

Dynamin-like guanylate binding protein (GBP)−1 (GBP1) is a GTPase that has inducible expression in immune cells and tonic expression in glandular and epidermal cells (71). It is a member of a class of interferon-inducible antimicrobial proteins that bind to Gram-negative bacteria (71, 72). Specifically, GBP1 associates with *Shigella flexneri* and *Burkholderia thailandensis* and prevents their replication and spread by inhibiting actin-based motility. It was shown that this occurs through the interaction of GBP1 with the O-antigen portion of lipopolysaccharides, as GBP1 is much less effective against *S. flexneri* without O-antigen (“rough” polysaccharide) (72). Nod-like receptors (NLRs) are intracellular pattern recognition receptors whose ligand specificities are determined by leucine-rich repeat and an NH_2_-terminal effector domain. They are critical to defense against pathogens through their activation of inflammasomes, autophagy, inflammatory signaling, and antigen presentation. Variation in NLR expression may lead to susceptibility to infectious disease, such as HIV-1, and autoimmune disorders, such as inflammatory bowel disease and psoriasis. Expression varies by receptor, but they can be found in most innate immune cells, epithelial cells, keratinocytes, and hepatocytes . While evidence for NLRs binding to lipid molecules is much more limited, NLRP7 is activated by acylated bacterial lipopeptides produced by *Mycoplasma spp., S. aureus,* and *Listeria monocytogenes*. In collaboration with TLR2, activation of NLRP7 promotes IL-1ß secretion through caspase-1, potentially influencing the response to intracellular bacteria (73).

## MICROBIAL-DERIVED LIPIDS

Microbes are made up of numerous and diverse lipids, with some being uniquely present in certain microbial species (e.g., mycolic acid, lipopolysaccharide). One of these unique lipids is a derivative of cholesterol, called sterylglycoside and it is expressed by plants, algae, and fungi. In the context of fungal infections, exogenous sterylglycoside acts as a protective factor, modulating the host immune system through the promotion of CD4 +T cells in mice infected with *Cryptococcus neoformans,* an opportunistic pathogen that can cause life-threatening disease in the immunocompromised (74, 75). However, whether fungal-derived sterylglycoside interacts directly with immune cells is not known. Glycosylceramide is a sphingolipid expressed broadly with essential implications in fungal morphogenesis, virulence, and growth, among others. In contrast to SG, the expression of glycosylceramide enables the growth of *Cryptococcus neoformans*. Yeast that lack the enzyme required to produce glycosylceramide have a defect in their ability to grow extracellularly, particularly in the vasculature (76), and antibodies that target glycosylceramide are protective against lethal infection (77). Candidiasis, caused by the yeast *Candida albicans*, is a leading cause of nosocomial and opportunistic infections. Ergosterol is the principal sterol produced by *C. albicans,* and it has been shown to promote biofilm formation, as the administration of simvastatin ex vivo significantly inhibits biofilm formation in both lab and clinical isolates (78).

*Mycobacterium spp*. are amongst the most unique in their lipid composition, which contributes to the selective permeability of pharmaceuticals and resistance to antimicrobials. The predominant lipid species that constitute the *Mycobacterium* inner membrane (~42%) is diacyl phosphatidyldimannoside (Ac_2_PIM_2_), a precursor to lipomannan (LM) and lipoarabinomannan (LAM). Other PIMs, such as AcPIM_2_, AcPIM_4_/Ac_2_PIM_4_, and AcPIM_6_/Ac_2_PIM_6_, were also identified as inner membrane components. Glycopeptidolipids are a significant component of the outer cell membrane, along with diacylglycerols, triacylglycerols, and free mycolic acids (79). Interestingly, deletion of a two-gene operon (*lprG-mfs*) in *Mycobacterium smegmatis* leads to increased drug permeability as these highly conserved transporters contribute to lipid transport and proper assembly of the mycobacterial outer membrane (80). Lipomannan, expressed on the mycobacterial cell wall, induces macrophage apoptosis and the production of IL-12 through TLR2, whereas lipoarabinomannan may protect infected and bystander cells from apoptosis through stabilization of Bcl-2 and reduction of IL-12 and TNF-a, the latter of which promotes bacterial replication (81). LphQ is a 19 kDa lipoprotein expressed by *Mycobacterium tuberculosis* that represses the expression of macrophage MHCII and induces apoptosis through both intrinsic (caspase-9) and extrinsic (AIF) mechanisms, both occurring in a TLR2-dependent manner (82–84). Finally, phthiocerol dimycocerosate, is a long-chain non-polar lipid expressed in the mycobacterial cell wall and promotes its pathogenesis through disruption of LC3-mediated phagocytosis, an essential mechanism for the clearance of intracellular pathogens (85).

Other Gram-positive bacteria express glycolipids and phospholipids, both attached to a diacylglycerol backbone. Interestingly, Gram-positive bacteria tend to amino-acylate their lipids, which masks the negative charge of the membrane’s extracellular surface to avoid recognition by cationic antimicrobial peptides (CAMPs), reduce the effect of antibiotics, and avoid killing by opsonophagocytosis (86, 87). Group B *Streptococcus* D-alanylates lipotechoic acid, which directly affects the effectivity of CAMPs, reducing their lytic effect. However, resistance to CAMPs was not due to changes in cell wall charge but rather the density of the cell wall (88). A recently discovered membrane glycolipid, Lys-Glc-DAG, is expressed in *Streptococcus agalactiae* and contributes to its pathogenesis through enhanced invasion of the blood brain barrier, however, through an unknown mechanism (89).

Gram-negative bacteria are surrounded by an inner and outer membrane, separated by a thin peptidoglycan layer. Their outer membrane comprises a non-symmetrical bilayer of lipopolysaccharides on the outside and phospholipids on the inside, and their inner membrane is composed of a standard phospholipid bilayer (90, 91). Lipid A, part of the lipopolysaccharide-containing outer layer, can be tetra-, penta-, hexta-, or hepta-acylated and is the primary cause of the severe response to endotoxin, eliciting both protective (anti-bacterial) and detrimental outcomes (endotoxin shock) (92). As described above and reviewed in detail elsewhere, extracellular lipid A is predominately sensed by TLR4 through coordination with LPS-binding protein, MD2, and CD14. However, immune-activating lipid molecules, such as lipid A and cardiolipin, can also be found in outer membrane vesicles secreted by Gram-negative bacteria, eliciting activation of TLR4 (69). In addition to lipid A, cardiolipins induce inflammatory signaling through interactions with TLR4. Cardiolipins are tetra-acylated diphosphatidylglycerols, found in both saturated and unsaturated forms, which determine their ability to induce TLR4 dimerization and activation (93). Saturated cardiolipin induces similar effects as lipid A, causing an increase in pro-inflammatory cytokine production. In contrast, unsaturated cardiolipin antagonizes TLR4 activation, presumably through competitive inhibition of lipid A, with higher levels of antagonism in shorter acyl side chains (93).

In low phosphorous environments, Gram-negative bacteria increase the production of amino acid-containing lipids rather than phospholipids, the most predominant of which is ornithine-lipid (OL). The switch from LPS to OL production leads to enhanced protection from pH stress and improved replication under phosphate-limited conditions (94). OL stimulates TLR4, albeit to a lesser degree than LPS, while promoting antibiotic resistance and chronic infection with *P. aeruginosa* (95). OL also activates the NLRP3 inflammasome while inhibiting LPS-induced non-canonical inflammasome activation and subsequently, IL-1B release (96). Gram-negative bacteria have additional surface-exposed lipoproteins, which play a prominent role in the cellular response to infection. *Burkholderia cenocepacia,* an opportunistic class of pathogens causing lung infections in cystic fibrosis patients, expresses a lipoprotein (Pal) that binds to DAP-type peptidoglycan but also mediates attachment and inflammation of human lung epithelial cells. While it is known that Pal stabilizes the bacterial cell wall, the host-specific binding partner of Pal has yet to be identified (97).

Instead of LPS, several pathogenic and non-pathogenic bacteria produce sphingolipids similar to eukaryotes, though structurally more varied (98). *Porphyromonas gingivalis*, an important oral pathogen, expresses membrane-associated dihydroceramides that contribute to cellular structure, capsule production, production of virulence factors, and resistance to oxidative stress (99). *Sphingomonas* spp. of bacteria produce glycosphingolipids instead of lipopolysaccharides, which activate immune signaling independently of TLR4. Instead, glycosphingolipids are recognized by NKT cells through CD1d, leading to the production of IL-4, IFN-g, and clearance of the bacteria from the liver (98, 100). Glycosphingolipids are not only present in pathogenic bacteria but are also found in common commensals. *Bacteroides* spp. of bacteria, a common occupant of the gut microbiota, produces α-Galactosylceramide which is another ligand for CD1d on NKT cells, suggesting a role for CD1d/α-GalCer interactions during immune homeostasis in the gut (101). Bacterial-derived fatty acid molecules, some of which are sphingolipid-derived (e.g., S1P), influence immunity and homeostasis. In fact, bacteria-derived *N*-acyl amides interact with host G-protein coupled receptors (GPCRs), including *N*-palmitoyl serinol with GPR119, *N*-3-hydroxypalmitoyl ornithine with S1PR4, and *N*-myristoyl alanine with G2A, leading to regulation of glucose (102).

As mentioned above, many bacterial species produce lipid-rich OMVs to deliver cargo to target cells, often as a mechanism of virulence (69). *Campylobacter jejuni,* a prominent cause of gastroenteritis and diarrheal disease, lacks secretion systems commonly used by other bacteria. As such, *C. jejuni* uses OMVs to deliver immune-evading and cytotoxic cargo to cells by integrating with cholesterol-containing lipid rafts (103). Specifically, *C. jejuni* OMVs contain a number of glycoproteins that may be used for immune evasion; however, OMVs were also shown to elicit inflammatory cytokine expression and cytotoxicity in intestinal epithelial cells (103). While Gram-negative bacteria are well-known OMV producers, delivering a variety of cargo to target cells, OMVs are much less well-characterized in Gram-positive bacteria. However, more recent studies have shown that Gram-positive bacteria also produce OMVs, including *S. aureus, B. anthracis,* and *Streptococcus spp. S. pneumoniae,* an important lung pathogen with a number of immune evasion techniques, produces OMVs rich in lipoproteins and short chain fatty acids. Spn-derived OMVs have broad impacts on immune function, modifying T-cell and NK-cell numbers, along with the phenotype of lung macrophages. Here, Spn-derived OMVs promote an M2-like phenotype in lung macrophages, allowing them to become a reservoir for infection (104). In addition to bacteria, fungal pathogens deliver cargo to host cells via sterol- and GlyCer-rich outer membrane vesicles (105, 106). In the context of *C. neoformans,* OMVs are produced, which modulate the function of macrophages, stimulating nitric oxide, cytokine, and antimicrobial protein production and phagocytic capacity (107), potentially resulting from virulence factor delivery (108).

## IMPLICATIONS FOR INFECTION

Microbial lipid-host cell receptor interactions positively and negatively impact host response and recovery from infection . As discussed above, lipid molecules comprise a substantial portion of the extracellular surface of bacterial, fungal, and parasitic pathogens (Table 2). As such, they provide a permeability barrier to host and synthetically-derived antimicrobial compounds. *Mycobacterium tuberculosis* has proven to be one of the most highly challenging pathogens to develop effective therapeutics for, due to the complex nature of its cell wall structure, rendering it relatively impermeable. *M. tuberculosis* also introduces modifications to the lipid structure of the cell wall to become more resistant to cationic antimicrobial peptides produced by the host. In addition to modifications discussed in the previous section, LysX is a two-domain protein produced by *M. tuberculosis* that lysinylates cell wall phospholipids, such as phosphatidylglycerol, rendering the organism less susceptible to killing. As evidence of this, *lysX* mutant TB strains activate higher cytokine expression levels in macrophages *in vitro* and exhibit less growth *in vivo*, with improved host pathology (109). On the host side, two single nucleotide polymorphisms have been discovered in the scavenger receptor, Marco, that leads to impaired phagocytosis and increased risk and severity of pulmonary TB, including chest X-ray abnormalities. Intriguingly, polymorphisms were also associated with genetic variation in the bacterium, specifically the modern Beijing strain of *M. tuberculosis* (59).

As described previously, phthiocerol dimycocerosate (PDIM), is a unique outer membrane lipid that serves as a virulence factor for *M. tuberculosis*. PDIM also contributes to antibiotic resistance, where transposon libraries revealed that deficiencies in PDIM promote antibiotic susceptibility in nutrient-limited conditions (110). Drug-resistant *M. tuberculosis* also has higher PDIM:PIM ratios, which increase the hydrophobicity of its cell wall, along with alkylforms of PDIM that do not exist in drug-susceptible *M. tuberculosis*. These modifications correlated with decreased uptake in human monocyte-derived macrophages and increased intracellular growth (111). As current treatment options are limited and lengthy, requiring six months of rifampin (RIF), isoniazid (INH), ethambutol (EMB), and pyrazinamide (PZA) (112), it will be critical to determine the cause and solutions to antimicrobial resistance in *M. tuberculosis*.

Lipopolysaccharide, the predominant glycolipid on the outer membrane of Gram-negative bacteria, elicits protective immunity as TLR4 KO mice are hyporesponsive to LPS and are significantly more susceptible to various infections, including those that are independent of Gram-negative bacteria (113–115). However, timing, duration, and type of LPS can tip the scales of protection towards endotoxemia and uncontrolled inflammatory signaling, resulting in disseminated intravascular coagulation, organ failure, and death. While the protective impact of TLR4 against infection has been known for some time, recent data has shown that TLR4/LPS signaling induces neutrophil extracellular trap formation and variations in O-antigen can impact susceptibility to bacterial killing by NETs, where uropathogenic *E. coli* that expresses O-antigen exhibits higher levels of NET formation but also increased resistance to killing (116).

LPS activation of TLR4 at the site of infection is a generally beneficial response, leading to pathogen clearance; however, if the infection is not sufficiently controlled, bacteria and LPS can make their way into circulation, causing sepsis and/or endotoxemia. Endotoxemia is an extremely dangerous condition causing acute kidney injury, hepatic dysfunction, disseminated intravascular coagulation, and shock. With levels of endotoxin activity level greater than 0.9, there is a greater than 80% chance of mortality if left untreated (117). Risk factors for sepsis and severe endotoxemia are active lines of investigation. Recently, the histone acetyltransferase, CREB-binding protein (CBP), was shown to be closely associated with TLR4. Here, the TIR domain of TLR4 is acetylated, which increases TLR4 signaling through the MAL adaptor protein. Ablation of the acetylation site improved survival while limiting inflammation in a mouse model of sepsis. Moreover, CD16 +monocytes isolated from patients with sepsis had high levels of TLR4-TIR acetylation that was associated with a pro-inflammatory gene profile (118).

*Pseudomonas aeruginosa,* an opportunistic Gram-negative pathogen that is a frequent cause of lung infections in patients with cystic fibrosis, can vary the structure of their lipid A molecule in the cystic fibrosis lung such that immunity is altered. PagL-mediated lipid A deacylation is lowered during acute infection with *Pseudomonas*, resulting in increased cytokine signaling in Thp-1 cells and primary macrophages (119). In contrast, mutants that lack hydroxylation at LpxO2-specific lipid A sites, a mutation that has been shown to occur during chronic CF infection (120), inhibit TLR4/MD-2-dependent cytokine signaling, with surprisingly little change in the severity of infection in mice (119). Finally, as mentioned previously, outer membrane vesicles are lipid-rich vesicles that deliver cargo to target cells during infection. In the context of *Cryptococcus neoformans* infection, outer membrane vesicles increase the fungal burden in the brain, resulting in cryptococcal meningitis.

## FUTURE DIRECTIONS/CONCLUSION

While specific lipid interactions, such as TLR4/LPS, have been studied in great detail, there are many under-characterized lipid-receptor interactions that may be beneficial for the development of therapeutics or aid in vaccine design. As many clever pathogens can modify lipid moieties to avoid immune detection and aid in antibiotic resistance, staying vigilant of evolving lipid modification strategies will be imperative. Moreover, there is still much to be discovered regarding the host response to lipid ligands. While often beneficial, certain lipid-receptor interactions can have devastating consequences depending on the location and duration of signaling. As such, limiting these interactions may be equally therapeutic in certain contexts, such as in sepsis. Finally, the majority of lipid research has focused on bacterial infections, while many other pathogenic microorganisms, including viruses, fungi, and parasites, use unique lipids as mechanisms of virulence. Utilizing what we have discovered regarding bacterial pathogens, future research should expand to more diverse species with limited treatment options.

## References

[1] Chandler CE, Ernst RK. 2017. Bacterial lipids: powerful modifiers of the innate immune response. F1000Res 6:6. doi:10.12688/f1000research.11388.1 PMC5553087 28868130

[2] Mazzon M, Mercer J. 2014. Lipid interactions during virus entry and infection. Cell Microbiol 16:1493–1502. doi:10.1111/cmi.12340 25131438 PMC4265854

[3] Cartier A, Hla T. 2019. Sphingosine 1-phosphate: lipid signaling in pathology and therapy. Science 366:eaar5551. doi:10.1126/science.aar5551 31624181 PMC7661103

[4] Liu W-C, Yang Y-H, Wang Y-C, Chang W-M, Wang C-W. 2023. Maresin: macrophage mediator for resolving inflammation and bridging tissue regeneration-a system-based preclinical systematic review. Int J Mol Sci 24:13. doi:10.3390/ijms241311012 PMC10341548 37446190

[5] Sheppe AEF, Edelmann MJ. 2021. Roles of eicosanoids in regulating inflammation and neutrophil migration as an innate host response to bacterial infections. Infect Immun 89:e0009521. doi:10.1128/IAI.00095-21 34031130 PMC8281227

[6] Fitzgerald KA, Kagan JC. 2020. Toll-like receptors and the control of immunity. Cell 180:1044–1066. doi:10.1016/j.cell.2020.02.041 32164908 PMC9358771

[7] Muzio M, Bosisio D, Polentarutti N, D’amico G, Stoppacciaro A, Mancinelli R, van’t Veer C, Penton-Rol G, Ruco LP, Allavena P, Mantovani A. 2000. Differential expression and regulation of toll-like receptors (TLR) in human leukocytes: selective expression of TLR3 in dendritic cells. J Immunol 164:5998–6004. doi:10.4049/jimmunol.164.11.5998 10820283

[8] Akira S, Takeda K, Kaisho T. 2001. Toll-like receptors: critical proteins linking innate and acquired immunity. Nat Immunol 2:675–680. doi:10.1038/90609 11477402

[9] Kawai T, Ikegawa M, Ori D, Akira S. 2024. Decoding Toll-like receptors: recent insights and perspectives in innate immunity. Immunity 57:649–673. doi:10.1016/j.immuni.2024.03.004 38599164

[10] Duan T, Du Y, Xing C, Wang HY, Wang R-F. 2022. Toll-like receptor signaling and its role in cell-mediated immunity. Front Immunol 13:812774. doi:10.3389/fimmu.2022.812774 35309296 PMC8927970

[11] Zhang S-Y, Jouanguy E, Ugolini S, Smahi A, Elain G, Romero P, Segal D, Sancho-Shimizu V, Lorenzo L, Puel A, et al.. 2007. TLR3 deficiency in patients with herpes simplex encephalitis. Science 317:1522–1527. doi:10.1126/science.1139522 17872438

[12] Picard C, von Bernuth H, Ghandil P, Chrabieh M, Levy O, Arkwright PD, McDonald D, Geha RS, Takada H, Krause JC, et al.. 2010. Clinical features and outcome of patients with IRAK-4 and MyD88 deficiency. Medicine (Baltimore) 89:403–425. doi:10.1097/MD.0b013e3181fd8ec3 21057262 PMC3103888

[13] Maglione PJ, Simchoni N, Black S, Radigan L, Overbey JR, Bagiella E, Bussel JB, Bossuyt X, Casanova J-L, Meyts I, Cerutti A, Picard C, Cunningham-Rundles C. 2014. IRAK-4 and MyD88 deficiencies impair IgM responses against T-independent bacterial antigens. Blood 124:3561–3571. doi:10.1182/blood-2014-07-587824 25320238 PMC4256908

[14] Cook DN, Pisetsky DS, Schwartz DA. 2004. Toll-like receptors in the pathogenesis of human disease. Nat Immunol 5:975–979. doi:10.1038/ni1116 15454920

[15] Kang S-S, Noh SY, Park O-J, Yun C-H, Han SH. 2015. Staphylococcus aureus induces IL-8 expression through its lipoproteins in the human intestinal epithelial cell, Caco-2. Cytokine 75:174–180. doi:10.1016/j.cyto.2015.04.017 25982554

[16] Abreu MT. 2010. Toll-like receptor signalling in the intestinal epithelium: how bacterial recognition shapes intestinal function. Nat Rev Immunol 10:131–144. doi:10.1038/nri2707 20098461

[17] Kurokawa K, Lee H, Roh K-B, Asanuma M, Kim YS, Nakayama H, Shiratsuchi A, Choi Y, Takeuchi O, Kang HJ, Dohmae N, Nakanishi Y, Akira S, Sekimizu K, Lee BL. 2009. The triacylated ATP binding cluster transporter substrate-binding lipoprotein of Staphylococcus aureus functions as a native ligand for Toll-like receptor 2. J Biol Chem 284:8406–8411. doi:10.1074/jbc.M809618200 19139093 PMC2659198

[18] Kim NJ, Ahn KB, Jeon JH, Yun C-H, Finlay BB, Han SH. 2015. Lipoprotein in the cell wall of Staphylococcus aureus is a major inducer of nitric oxide production in murine macrophages. Mol Immunol 65:17–24. doi:10.1016/j.molimm.2014.12.016 25600878

[19] Lu YC, Yeh WC, Ohashi PS. 2008. LPS/TLR4 signal transduction pathway. Cytokine 42:145–151. doi:10.1016/j.cyto.2008.01.006 18304834

[20] Park BS, Lee JO. 2013. Recognition of lipopolysaccharide pattern by TLR4 complexes. Exp Mol Med 45:e66. doi:10.1038/emm.2013.97 24310172 PMC3880462

[21] Sancho D, Reis e Sousa C. 2012. Signaling by myeloid C-type lectin receptors in immunity and homeostasis. Annu Rev Immunol 30:491–529. doi:10.1146/annurev-immunol-031210-101352 22224766 PMC4480235

[22] Drouin M, Saenz J, Chiffoleau E. 2020. C-type lectin-like receptors: head or tail in cell death immunity. Front Immunol 11:251. doi:10.3389/fimmu.2020.00251 32133013 PMC7040094

[23] Akhmedov A, Sawamura T, Chen C-H, Kraler S, Vdovenko D, Lüscher TF. 2021. Lectin-like oxidized low-density lipoprotein receptor-1 (LOX-1): a crucial driver of atherosclerotic cardiovascular disease. Eur Heart J 42:1797–1807. doi:10.1093/eurheartj/ehaa770 33159784

[24] Geijtenbeek TBH, Gringhuis SI. 2009. Signalling through C-type lectin receptors: shaping immune responses. Nat Rev Immunol 9:465–479. doi:10.1038/nri2569 19521399 PMC7097056

[25] Chen S-T, Lin Y-L, Huang M-T, Wu M-F, Cheng S-C, Lei H-Y, Lee C-K, Chiou T-W, Wong C-H, Hsieh S-L. 2008. CLEC5A is critical for dengue-virus-induced lethal disease. Nature 453:672–676. doi:10.1038/nature07013 18496526

[26] Reis e Sousa C, Yamasaki S, Brown GD. 2024. Myeloid C-type lectin receptors in innate immune recognition. Immunity 57:700–717. doi:10.1016/j.immuni.2024.03.005 38599166

[27] Lu X, Nagata M, Yamasaki S. 2018. Mincle: 20 years of a versatile sensor of insults. Int Immunol 30:233–239. doi:10.1093/intimm/dxy028 29726997

[28] Ishikawa E, Ishikawa T, Morita YS, Toyonaga K, Yamada H, Takeuchi O, Kinoshita T, Akira S, Yoshikai Y, Yamasaki S. 2009. Direct recognition of the mycobacterial glycolipid, trehalose dimycolate, by C-type lectin Mincle. J Exp Med 206:2879–2888. doi:10.1084/jem.20091750 20008526 PMC2806462

[29] Feinberg H, Rambaruth NDS, Jégouzo SAF, Jacobsen KM, Djurhuus R, Poulsen TB, Weis WI, Taylor ME, Drickamer K. 2016. Binding sites for acylated trehalose analogs of glycolipid ligands on an extended carbohydrate recognition domain of the macrophage receptor mincle. J Biol Chem 291:21222–21233. doi:10.1074/jbc.M116.749515 27542410 PMC5076529

[30] Schoenen H, Bodendorfer B, Hitchens K, Manzanero S, Werninghaus K, Nimmerjahn F, Agger EM, Stenger S, Andersen P, Ruland J, Brown GD, Wells C, Lang R. 2010. Cutting edge: mincle is essential for recognition and adjuvanticity of the mycobacterial cord factor and its synthetic analog trehalose-dibehenate. J Immunol 184:2756–2760. doi:10.4049/jimmunol.0904013 20164423 PMC3442336

[31] Shenderov K, Barber DL, Mayer-Barber KD, Gurcha SS, Jankovic D, Feng CG, Oland S, Hieny S, Caspar P, Yamasaki S, Lin X, Ting JP-Y, Trinchieri G, Besra GS, Cerundolo V, Sher A. 2013. Cord factor and peptidoglycan recapitulate the Th17-promoting adjuvant activity of mycobacteria through mincle/CARD9 signaling and the inflammasome. J Immunol 190:5722–5730. doi:10.4049/jimmunol.1203343 23630357 PMC3719989

[32] Ishikawa T, Itoh F, Yoshida S, Saijo S, Matsuzawa T, Gonoi T, Saito T, Okawa Y, Shibata N, Miyamoto T, Yamasaki S. 2013. Identification of distinct ligands for the C-type lectin receptors Mincle and Dectin-2 in the pathogenic fungus Malassezia. Cell Host Microbe 13:477–488. doi:10.1016/j.chom.2013.03.008 23601109

[33] Imai T, Matsumura T, Mayer-Lambertz S, Wells CA, Ishikawa E, Butcher SK, Barnett TC, Walker MJ, Imamura A, Ishida H, Ikebe T, Miyamoto T, Ato M, Ohga S, Lepenies B, van Sorge NM, Yamasaki S. 2018. Lipoteichoic acid anchor triggers Mincle to drive protective immunity against invasive group A Streptococcus infection. Proc Natl Acad Sci U S A 115:E10662–E10671. doi:10.1073/pnas.1809100115 30352847 PMC6233082

[34] Behler-Janbeck F, Takano T, Maus R, Stolper J, Jonigk D, Tort Tarrés M, Fuehner T, Prasse A, Welte T, Timmer MSM, Stocker BL, Nakanishi Y, Miyamoto T, Yamasaki S, Maus UA. 2016. C-type lectin mincle recognizes glucosyl-diacylglycerol of Streptococcus pneumoniae and plays a protective role in pneumococcal pneumonia. PLoS Pathog 12:e1006038. doi:10.1371/journal.ppat.1006038 27923071 PMC5140071

[35] Monteiro MA, Chan KH, Rasko DA, Taylor DE, Zheng PY, Appelmelk BJ, Wirth HP, Yang M, Blaser MJ, Hynes SO, Moran AP, Perry MB. 1998. Simultaneous expression of type 1 and type 2 Lewis blood group antigens by Helicobacter pylori lipopolysaccharides. Molecular mimicry between h. pylori lipopolysaccharides and human gastric epithelial cell surface glycoforms. J Biol Chem 273:11533–11543. doi:10.1074/jbc.273.19.11533 9565568

[36] Bergman MP, Engering A, Smits HH, van Vliet SJ, van Bodegraven AA, Wirth H-P, Kapsenberg ML, Vandenbroucke-Grauls CMJE, van Kooyk Y, Appelmelk BJ. 2004. Helicobacter pylori modulates the T helper cell 1/T helper cell 2 balance through phase-variable interaction between lipopolysaccharide and DC-SIGN. J Exp Med 200:979–990. doi:10.1084/jem.20041061 15492123 PMC2211851

[37] Haque M, Hirai Y, Yokota K, Mori N, Jahan I, Ito H, Hotta H, Yano I, Kanemasa Y, Oguma K. 1996. Lipid profile of Helicobacter spp.: presence of cholesteryl glucoside as a characteristic feature. J Bacteriol 178:2065–2070. doi:10.1128/jb.178.7.2065-2070.1996 8606185 PMC177906

[38] Jan H-M, Chen Y-C, Shih Y-Y, Huang Y-C, Tu Z, Ingle AB, Liu S-W, Wu M-S, Gervay-Hague J, Mong K-KT, Chen Y-R, Lin C-H. 2016. Metabolic labelling of cholesteryl glucosides in Helicobacter pylori reveals how the uptake of human lipids enhances bacterial virulence. Chem Sci 7:6208–6216. doi:10.1039/c6sc00889e 30034762 PMC6024656

[39] Nagata M, Toyonaga K, Ishikawa E, Haji S, Okahashi N, Takahashi M, Izumi Y, Imamura A, Takato K, Ishida H, Nagai S, Illarionov P, Stocker BL, Timmer MSM, Smith DGM, Williams SJ, Bamba T, Miyamoto T, Arita M, Appelmelk BJ, Yamasaki S. 2021. Helicobacter pylori metabolites exacerbate gastritis through C-type lectin receptors. J Exp Med 218:e20200815. doi:10.1084/jem.20200815 32991669 PMC7527975

[40] He Y-X, Ye C-L, Zhang P, Li Q, Park CG, Yang K, Jiang L-Y, Lv Y, Ying X-L, Ding H-H, et al.. 2019. Yersinia pseudotuberculosis exploits CD209 receptors for promoting host dissemination and infection. Infect Immun 87:e00654-18. doi:10.1128/IAI.00654-18 30348825 PMC6300620

[41] Saijo S, Iwakura Y. 2011. Dectin-1 and Dectin-2 in innate immunity against fungi. Int Immunol 23:467–472. doi:10.1093/intimm/dxr046 21677049

[42] Wittmann A, Lamprinaki D, Bowles KM, Katzenellenbogen E, Knirel YA, Whitfield C, Nishimura T, Matsumoto N, Yamamoto K, Iwakura Y, Saijo S, Kawasaki N. 2016. Dectin-2 recognizes mannosylated O-antigens of human opportunistic pathogens and augments lipopolysaccharide activation of myeloid cells. J Biol Chem 291:17629–17638. doi:10.1074/jbc.M116.741256 27358401 PMC5016159

[43] Omahdi Z, Horikawa Y, Nagae M, Toyonaga K, Imamura A, Takato K, Teramoto T, Ishida H, Kakuta Y, Yamasaki S. 2020. Structural insight into the recognition of pathogen-derived phosphoglycolipids by C-type lectin receptor DCAR. J Biol Chem 295:5807–5817. doi:10.1074/jbc.RA120.012491 32139512 PMC7186165

[44] Toyonaga K, Torigoe S, Motomura Y, Kamichi T, Hayashi JM, Morita YS, Noguchi N, Chuma Y, Kiyohara H, Matsuo K, Tanaka H, Nakagawa Y, Sakuma T, Ohmuraya M, Yamamoto T, Umemura M, Matsuzaki G, Yoshikai Y, Yano I, Miyamoto T, Yamasaki S. 2016. C-type lectin receptor DCAR recognizes mycobacterial phosphatidyl-inositol mannosides to promote a Th1 response during infection. Immunity 45:1245–1257. doi:10.1016/j.immuni.2016.10.012 27887882

[45] Canton J, Neculai D, Grinstein S. 2013. Scavenger receptors in homeostasis and immunity. Nat Rev Immunol 13:621–634. doi:10.1038/nri3515 23928573

[46] Huang L, Chambliss KL, Gao X, Yuhanna IS, Behling-Kelly E, Bergaya S, Ahmed M, Michaely P, Luby-Phelps K, Darehshouri A, Xu L, Fisher EA, Ge W-P, Mineo C, Shaul PW. 2019. SR-B1 drives endothelial cell LDL transcytosis via DOCK4 to promote atherosclerosis. Nature 569:565–569. doi:10.1038/s41586-019-1140-4 31019307 PMC6631346

[47] Dieudonné A, Torres D, Blanchard S, Taront S, Jeannin P, Delneste Y, Pichavant M, Trottein F, Gosset P. 2012. Scavenger receptors in human airway epithelial cells: role in response to double-stranded RNA. PLoS One 7:e41952. doi:10.1371/journal.pone.0041952 22879901 PMC3413698

[48] Kattoor AJ, Goel A, Mehta JL. 2019. LOX-1: Regulation, signaling and its role in atherosclerosis. Antioxidants (Basel) 8:218. doi:10.3390/antiox8070218 31336709 PMC6680802

[49] Kzhyshkowska J, Neyen C, Gordon S. 2012. Role of macrophage scavenger receptors in atherosclerosis. Immunobiology 217:492–502. doi:10.1016/j.imbio.2012.02.015 22437077

[50] Park YM. 2014. CD36, a scavenger receptor implicated in atherosclerosis. Exp Mol Med 46:e99. doi:10.1038/emm.2014.38 24903227 PMC4081553

[51] Chen Y, Zhang J, Cui W, Silverstein RL. 2022. CD36, a signaling receptor and fatty acid transporter that regulates immune cell metabolism and fate. J Exp Med 219:e20211314. doi:10.1084/jem.20211314 35438721 PMC9022290

[52] Hoebe K, Georgel P, Rutschmann S, Du X, Mudd S, Crozat K, Sovath S, Shamel L, Hartung T, Zähringer U, Beutler B. 2005. CD36 is a sensor of diacylglycerides. Nature 433:523–527. doi:10.1038/nature03253 15690042

[53] Jimenez-Dalmaroni MJ, Xiao N, Corper AL, Verdino P, Ainge GD, Larsen DS, Painter GF, Rudd PM, Dwek RA, Hoebe K, Beutler B, Wilson IA. 2009. Soluble CD36 ectodomain binds negatively charged diacylglycerol ligands and acts as a co-receptor for TLR2. PLoS One 4:e7411. doi:10.1371/journal.pone.0007411 19847289 PMC2760212

[54] Sharif O, Matt U, Saluzzo S, Lakovits K, Haslinger I, Furtner T, Doninger B, Knapp S. 2013. The scavenger receptor CD36 downmodulates the early inflammatory response while enhancing bacterial phagocytosis during pneumococcal pneumonia. J Immunol 190:5640–5648. doi:10.4049/jimmunol.1202270 23610144

[55] Józefowski S, Sobota A, Pawłowski A, Kwiatkowska K. 2011. Mycobacterium tuberculosis lipoarabinomannan enhances LPS-induced TNF-α production and inhibits NO secretion by engaging scavenger receptors. Microb Pathog 50:350–359. doi:10.1016/j.micpath.2011.03.001 21419839

[56] Baranova IN, Kurlander R, Bocharov AV, Vishnyakova TG, Chen Z, Remaley AT, Csako G, Patterson AP, Eggerman TL. 2008. Role of human CD36 in bacterial recognition, phagocytosis, and pathogen-induced JNK-mediated signaling. J Immunol 181:7147–7156. doi:10.4049/jimmunol.181.10.7147 18981136 PMC3842223

[57] Wang J, Cao H, Yang H, Wang N, Weng Y, Luo H. 2024. The function of CD36 in Mycobacterium tuberculosis infection. Front Immunol 15:1413947. doi:10.3389/fimmu.2024.1413947 38881887 PMC11176518

[58] Xing Q, Feng Y, Sun H, Yang S, Sun T, Guo X, Ji F, Wu B, Zhou D. 2021. Scavenger receptor MARCO contributes to macrophage phagocytosis and clearance of tumor cells. Exp Cell Res 408:112862. doi:10.1016/j.yexcr.2021.112862 34626585

[59] Thuong NTT, Tram TTB, Dinh TD, Thai PVK, Heemskerk D, Bang ND, Chau TTH, Russell DG, Thwaites GE, Hawn TR, Caws M, Dunstan SJ. 2016. MARCO variants are associated with phagocytosis, pulmonary tuberculosis susceptibility and Beijing lineage. Genes Immun 17:419–425. doi:10.1038/gene.2016.43 27853145 PMC5133378

[60] Plüddemann A, Mukhopadhyay S, Sankala M, Savino S, Pizza M, Rappuoli R, Tryggvason K, Gordon S. 2009. SR-A, MARCO and TLRs differentially recognise selected surface proteins from Neisseria meningitidis: an example of fine specificity in microbial ligand recognition by innate immune receptors. J Innate Immun 1:153–163. doi:10.1159/000155227 20375573 PMC7312862

[61] Wei C, Wan L, Yan Q, Wang X, Zhang J, Yang X, Zhang Y, Fan C, Li D, Deng Y, et al.. 2020. HDL-scavenger receptor B type 1 facilitates SARS-CoV-2 entry. Nat Metab 2:1391–1400. doi:10.1038/s42255-020-00324-0 33244168

[62] Cohen NR, Garg S, Brenner MB. 2009. Antigen presentation by CD1 lipids, T cells, and NKT cells in microbial immunity. Adv Immunol 102:1–94. doi:10.1016/S0065-2776(09)01201-2 19477319

[63] Singh AK, Tripathi P, Cardell SL. 2018. Type II NKT cells: an elusive population with immunoregulatory properties. Front Immunol 9:1969. doi:10.3389/fimmu.2018.01969 30210505 PMC6120993

[64] Tatituri RVV, Watts GFM, Bhowruth V, Barton N, Rothchild A, Hsu F-F, Almeida CF, Cox LR, Eggeling L, Cardell S, Rossjohn J, Godfrey DI, Behar SM, Besra GS, Brenner MB, Brigl M. 2013. Recognition of microbial and mammalian phospholipid antigens by NKT cells with diverse TCRs. Proc Natl Acad Sci U S A 110:1827–1832. doi:10.1073/pnas.1220601110 23307809 PMC3562825

[65] Wolf BJ, Tatituri RVV, Almeida CF, Le Nours J, Bhowruth V, Johnson D, Uldrich AP, Hsu F-F, Brigl M, Besra GS, Rossjohn J, Godfrey DI, Brenner MB. 2015. Identification of a potent microbial lipid antigen for diverse NKT cells. J Immunol 195:2540–2551. doi:10.4049/jimmunol.1501019 26254340 PMC5030721

[66] Jun H-K, Jung Y-J, Ji S, An S-J, Choi B-K. 2018. Caspase-4 activation by a bacterial surface protein is mediated by cathepsin G in human gingival fibroblasts. Cell Death Differ 25:380–391. doi:10.1038/cdd.2017.167 29077095 PMC5762851

[67] Smith AP, Creagh EM. 2022. Caspase-4 and -5 biology in the pathogenesis of inflammatory bowel disease. Front Pharmacol 13:919567. doi:10.3389/fphar.2022.919567 35712726 PMC9194562

[68] Lagrange B, Benaoudia S, Wallet P, Magnotti F, Provost A, Michal F, Martin A, Di Lorenzo F, Py BF, Molinaro A, Henry T. 2018. Human caspase-4 detects tetra-acylated LPS and cytosolic Francisella and functions differently from murine caspase-11. Nat Commun 9:242. doi:10.1038/s41467-017-02682-y 29339744 PMC5770465

[69] Giordano NP, Cian MB, Dalebroux ZD. 2020. Outer membrane lipid secretion and the innate immune response to Gram-negative bacteria. Infect Immun 88:e00920-19. doi:10.1128/IAI.00920-19 32253250 PMC7309610

[70] Vanaja SK, Russo AJ, Behl B, Banerjee I, Yankova M, Deshmukh SD, Rathinam VAK. 2016. Bacterial outer membrane vesicles mediate cytosolic localization of LPS and caspase-11 activation. Cell 165:1106–1119. doi:10.1016/j.cell.2016.04.015 27156449 PMC4874922

[71] Tretina K, Park E-S, Maminska A, MacMicking JD. 2019. Interferon-induced guanylate-binding proteins: Guardians of host defense in health and disease. J Exp Med 216:482–500. doi:10.1084/jem.20182031 30755454 PMC6400534

[72] Piro AS, Hernandez D, Luoma S, Feeley EM, Finethy R, Yirga A, Frickel EM, Lesser CF, Coers J. 2017. Detection of cytosolic Shigella flexneri via a C-terminal triple-arginine motif of GBP1 inhibits actin-based motility. MBio 8:e01979-17. doi:10.1128/mBio.01979-17 29233899 PMC5727416

[73] Khare S, Dorfleutner A, Bryan NB, Yun C, Radian AD, de Almeida L, Rojanasakul Y, Stehlik C. 2012. An NLRP7-containing inflammasome mediates recognition of microbial lipopeptides in human macrophages. Immunity 36:464–476. doi:10.1016/j.immuni.2012.02.001 22361007 PMC3315380

[74] Coelho C, Farrer RA. 2020. Pathogen and host genetics underpinning cryptococcal disease. Adv Genet 105:1–66. doi:10.1016/bs.adgen.2020.02.001 32560785 PMC7612911

[75] Rella A, Mor V, Farnoud AM, Singh A, Shamseddine AA, Ivanova E, Carpino N, Montagna MT, Luberto C, Del Poeta M. 2015. Role of Sterylglucosidase 1 (Sgl1) on the pathogenicity of Cryptococcus neoformans: potential applications for vaccine development. Front Microbiol 6:836. doi:10.3389/fmicb.2015.00836 26322039 PMC4531891

[76] Rittershaus PC, Kechichian TB, Allegood JC, Merrill AH Jr, Hennig M, Luberto C, Del Poeta M. 2006. Glucosylceramide synthase is an essential regulator of pathogenicity of Cryptococcus neoformans. J Clin Invest 116:1651–1659. doi:10.1172/JCI27890 16741577 PMC1466548

[77] Rodrigues ML, Shi L, Barreto-Bergter E, Nimrichter L, Farias SE, Rodrigues EG, Travassos LR, Nosanchuk JD. 2007. Monoclonal antibody to fungal glucosylceramide protects mice against lethal Cryptococcus neoformans infection. Clin Vaccine Immunol 14:1372–1376. doi:10.1128/CVI.00202-07 17715331 PMC2168121

[78] Liu G, Vellucci VF, Kyc S, Hostetter MK. 2009. Simvastatin inhibits Candida albicans biofilm in vitro. Pediatr Res 66:600–604. doi:10.1203/PDR.0b013e3181bd5bf8 19707174

[79] Bansal-Mutalik R, Nikaido H. 2014. Mycobacterial outer membrane is a lipid bilayer and the inner membrane is unusually rich in diacyl phosphatidylinositol dimannosides. Proc Natl Acad Sci U S A 111:4958–4963. doi:10.1073/pnas.1403078111 24639491 PMC3977252

[80] Hohl M, Remm S, Eskandarian HA, Dal Molin M, Arnold FM, Hürlimann LM, Krügel A, Fantner GE, Sander P, Seeger MA. 2019. Increased drug permeability of a stiffened mycobacterial outer membrane in cells lacking MFS transporter Rv1410 and lipoprotein LprG. Mol Microbiol 111:1263–1282. doi:10.1111/mmi.14220 30742339 PMC6519032

[81] Wojtas B, Fijalkowska B, Wlodarczyk A, Schollenberger A, Niemialtowski M, Hamasur B, Pawlowski A, Krzyzowska M. 2011. Mannosylated lipoarabinomannan balances apoptosis and inflammatory state in mycobacteria-infected and uninfected bystander macrophages. Microb Pathog 51:9–21. doi:10.1016/j.micpath.2011.03.004 21440050

[82] Sánchez A, Espinosa P, García T, Mancilla R. 2012. The 19 kDa Mycobacterium tuberculosis lipoprotein (LpqH) induces macrophage apoptosis through extrinsic and intrinsic pathways: a role for the mitochondrial apoptosis-inducing factor. Clin Dev Immunol 2012:950503. doi:10.1155/2012/950503 23316255 PMC3536062

[83] Noss EH, Pai RK, Sellati TJ, Radolf JD, Belisle J, Golenbock DT, Boom WH, Harding CV. 2001. Toll-like receptor 2-dependent inhibition of macrophage class II MHC expression and antigen processing by 19-kDa lipoprotein of Mycobacterium tuberculosis. J Immunol 167:910–918. doi:10.4049/jimmunol.167.2.910 11441098

[84] López M, Sly LM, Luu Y, Young D, Cooper H, Reiner NE. 2003. The 19-kDa Mycobacterium tuberculosis protein induces macrophage apoptosis through Toll-like receptor-2. J Immunol 170:2409–2416. doi:10.4049/jimmunol.170.5.2409 12594264

[85] Mittal E, Prasad GVRK, Upadhyay S, Sadadiwala J, Olive AJ, Yang G, Sassetti CM, Philips JA. 2024. Mycobacterium tuberculosis virulence lipid PDIM inhibits autophagy in mice. Nat Microbiol 9:2970–2984. doi:10.1038/s41564-024-01797-5 39242815 PMC12097147

[86] Peschel A, Jack RW, Otto M, Collins LV, Staubitz P, Nicholson G, Kalbacher H, Nieuwenhuizen WF, Jung G, Tarkowski A, van Kessel KP, van Strijp JA. 2001. Staphylococcus aureus resistance to human defensins and evasion of neutrophil killing via the novel virulence factor MprF is based on modification of membrane lipids with l-lysine. J Exp Med 193:1067–1076. doi:10.1084/jem.193.9.1067 11342591 PMC2193429

[87] Joyce L.R., Doran KS. 2023. Gram-positive bacterial membrane lipids at the host-pathogen interface. PLoS Pathog 19:e1011026. doi:10.1371/journal.ppat.1011026 36602959 PMC9815629

[88] Saar-Dover R, Bitler A, Nezer R, Shmuel-Galia L, Firon A, Shimoni E, Trieu-Cuot P, Shai Y. 2012. D-alanylation of lipoteichoic acids confers resistance to cationic peptides in group B streptococcus by increasing the cell wall density. PLoS Pathog 8:e1002891. doi:10.1371/journal.ppat.1002891 22969424 PMC3435245

[89] Joyce Luke R, Manzer HS, da C Mendonça J, Villarreal R, Nagao PE, Doran KS, Palmer KL, Guan Z. 2022. Identification of a novel cationic glycolipid in Streptococcus agalactiae that contributes to brain entry and meningitis. PLoS Biol 20:e3001555. doi:10.1371/journal.pbio.3001555 35180210 PMC8893666

[90] Steimle A, Autenrieth IB, Frick JS. 2016. Structure and function: Lipid A modifications in commensals and pathogens. Int J Med Microbiol 306:290–301. doi:10.1016/j.ijmm.2016.03.001 27009633

[91] Duong F, Eichler J, Price A, Leonard MR, Wickner W. 1997. Biogenesis of the gram-negative bacterial envelope. Cell 91:567–573. doi:10.1016/s0092-8674(00)80444-4 9393850

[92] Kim S, Patel DS, Park S, Slusky J, Klauda JB, Widmalm G, Im W. 2016. Bilayer properties of lipid A from various Gram-negative bacteria. Biophys J 111:1750–1760. doi:10.1016/j.bpj.2016.09.001 27760361 PMC5071556

[93] Pizzuto M, Lonez C, Baroja-Mazo A, Martínez-Banaclocha H, Tourlomousis P, Gangloff M, Pelegrin P, Ruysschaert J-M, Gay NJ, Bryant CE. 2019. Saturation of acyl chains converts cardiolipin from an antagonist to an activator of Toll-like receptor-4. Cell Mol Life Sci 76:3667–3678. doi:10.1007/s00018-019-03113-5 31062071 PMC6697720

[94] Bedoya-Pérez LP, Aguilar-Vera A, Sánchez-Pérez M, Utrilla J, Sohlenkamp C. 2024. Enhancing Escherichia coli abiotic stress resistance through ornithine lipid formation. Appl Microbiol Biotechnol 108:288. doi:10.1007/s00253-024-13130-5 38587638 PMC11001654

[95] Kim S-K, Park S-J, Li X-H, Choi Y-S, Im D-S, Lee J-H. 2018. Bacterial ornithine lipid, a surrogate membrane lipid under phosphate-limiting conditions, plays important roles in bacterial persistence and interaction with host. Environ Microbiol 20:3992–4008. doi:10.1111/1462-2920.14430 30252196

[96] Pizzuto M, Hurtado-Navarro L, Molina-Lopez C, Soubhye J, Gelbcke M, Rodriguez-Lopez S, Ruysschaert J-M, Schroder K, Pelegrin P. 2024. Ornithine lipid is a partial TLR4 agonist and NLRP3 activator. Cell Rep 43:114788. doi:10.1016/j.celrep.2024.114788 39340778

[97] Dennehy R, Romano M, Ruggiero A, Mohamed YF, Dignam SL, Mujica Troncoso C, Callaghan M, Valvano MA, Berisio R, McClean S. 2017. The Burkholderia cenocepacia peptidoglycan-associated lipoprotein is involved in epithelial cell attachment and elicitation of inflammation. Cell Microbiol 19. doi:10.1111/cmi.12691 27886433

[98] Heaver SL, Johnson EL, Ley RE. 2018. Sphingolipids in host-microbial interactions. Curr Opin Microbiol 43:92–99. doi:10.1016/j.mib.2017.12.011 29328957

[99] Moye ZD, Valiuskyte K, Dewhirst FE, Nichols FC, Davey ME. 2016. Synthesis of sphingolipids impacts survival of Porphyromonas gingivalis and the presentation of surface polysaccharides. Front Microbiol 7:1919. doi:10.3389/fmicb.2016.01919 27965646 PMC5126122

[100] Kinjo Y, Wu D, Kim G, Xing G-W, Poles MA, Ho DD, Tsuji M, Kawahara K, Wong C-H, Kronenberg M. 2005. Recognition of bacterial glycosphingolipids by natural killer T cells. Nature 434:520–525. doi:10.1038/nature03407 15791257

[101] Wieland Brown LC, Penaranda C, Kashyap PC, Williams BB, Clardy J, Kronenberg M, Sonnenburg JL, Comstock LE, Bluestone JA, Fischbach MA. 2013. Production of α-galactosylceramide by a prominent member of the human gut microbiota. PLoS Biol 11:e1001610. doi:10.1371/journal.pbio.1001610 23874157 PMC3712910

[102] Cohen LJ, Esterhazy D, Kim S-H, Lemetre C, Aguilar RR, Gordon EA, Pickard AJ, Cross JR, Emiliano AB, Han SM, Chu J, Vila-Farres X, Kaplitt J, Rogoz A, Calle PY, Hunter C, Bitok JK, Brady SF. 2017. Commensal bacteria make GPCR ligands that mimic human signalling molecules. Nature 549:48–53. doi:10.1038/nature23874 28854168 PMC5777231

[103] Elmi A, Watson E, Sandu P, Gundogdu O, Mills DC, Inglis NF, Manson E, Imrie L, Bajaj-Elliott M, Wren BW, Smith DGE, Dorrell N. 2012. Campylobacter jejuni outer membrane vesicles play an important role in bacterial interactions with human intestinal epithelial cells. Infect Immun 80:4089–4098. doi:10.1128/IAI.00161-12 22966047 PMC3497446

[104] Yerneni SS, Werner S, Azambuja JH, Ludwig N, Eutsey R, Aggarwal SD, Lucas PC, Bailey N, Whiteside TL, Campbell PG, Hiller NL. 2021. Pneumococcal extracellular vesicles modulate host immunity. MBio 12:e0165721. doi:10.1128/mBio.01657-21 34253061 PMC8406339

[105] Albuquerque PC, Nakayasu ES, Rodrigues ML, Frases S, Casadevall A, Zancope-Oliveira RM, Almeida IC, Nosanchuk JD. 2008. Vesicular transport in Histoplasma capsulatum: an effective mechanism for trans-cell wall transfer of proteins and lipids in ascomycetes. Cell Microbiol 10:1695–1710. doi:10.1111/j.1462-5822.2008.01160.x 18419773 PMC2562661

[106] Rella A, Farnoud AM, Del Poeta M. 2016. Plasma membrane lipids and their role in fungal virulence. Prog Lipid Res 61:63–72. doi:10.1016/j.plipres.2015.11.003 26703191 PMC4733445

[107] Oliveira DL, Freire-de-Lima CG, Nosanchuk JD, Casadevall A, Rodrigues ML, Nimrichter L. 2010. Extracellular vesicles from Cryptococcus neoformans modulate macrophage functions. Infect Immun 78:1601–1609. doi:10.1128/IAI.01171-09 20145096 PMC2849392

[108] Rodrigues ML, Nakayasu ES, Oliveira DL, Nimrichter L, Nosanchuk JD, Almeida IC, Casadevall A. 2008. Extracellular vesicles produced by Cryptococcus neoformans contain protein components associated with virulence. Eukaryot Cell 7:58–67. doi:10.1128/EC.00370-07 18039940 PMC2224146

[109] Maloney E, Stankowska D, Zhang J, Fol M, Cheng Q-J, Lun S, Bishai WR, Rajagopalan M, Chatterjee D, Madiraju MV. 2009. The two-domain LysX protein of Mycobacterium tuberculosis is required for production of lysinylated phosphatidylglycerol and resistance to cationic antimicrobial peptides. PLoS Pathog 5:e1000534. doi:10.1371/journal.ppat.1000534 19649276 PMC2713425

[110] Block AM, Namugenyi SB, Palani NP, Brokaw AM, Zhang L, Beckman KB, Tischler AD. 2023. Mycobacterium tuberculosis requires the outer membrane lipid phthiocerol dimycocerosate for starvation-induced antibiotic tolerance. mSystems 8:e0069922. doi:10.1128/msystems.00699-22 36598240 PMC9948706

[111] Schami A, Islam MN, Wall M, Hicks A, Meredith R, Kreiswirth B, Mathema B, Belisle JT, Torrelles JB. 2024. Drug resistant Mycobacterium tuberculosis strains have altered cell envelope hydrophobicity that influences infection outcomes in human macrophages. Sci Rep 14:30840. doi:10.1038/s41598-024-81457-0 39730579 PMC11681083

[112] Kim H, Shin SJ. 2023. Revolutionizing control strategies against Mycobacterium tuberculosis infection through selected targeting of lipid metabolism. Cell Mol Life Sci 80:291. doi:10.1007/s00018-023-04914-5 37704889 PMC11072447

[113] Hoshino K, Takeuchi O, Kawai T, Sanjo H, Ogawa T, Takeda Y, Takeda K, Akira S. 1999. Cutting edge: Toll-like receptor 4 (TLR4)-deficient mice are hyporesponsive to lipopolysaccharide: evidence for TLR4 as the Lps gene product. J Immunol 162:3749–3752. 10201887

[114] Shinya K, Ito M, Makino A, Tanaka M, Miyake K, Eisfeld AJ, Kawaoka Y. 2012. The TLR4-TRIF pathway protects against H5N1 influenza virus infection. J Virol 86:19–24. doi:10.1128/JVI.06168-11 22031950 PMC3255869

[115] Branger J, Leemans JC, Florquin S, Weijer S, Speelman P, Van Der Poll T. 2004. Toll-like receptor 4 plays a protective role in pulmonary tuberculosis in mice. Int Immunol 16:509–516. doi:10.1093/intimm/dxh052 14978024

[116] Lin W-H, Sheu S-M, Wu C-F, Huang W-C, Hsu L-J, Yu K-C, Cheng H-C, Kao C-Y, Wu J-J, Wang M-C, Teng C-H. 2025. O-antigen of uropathogenic Escherichia coli is required for induction of neutrophil extracellular traps. J Microbiol Immunol Infect 58:209–218. doi:10.1016/j.jmii.2024.12.007 39725572

[117] Kellum JA, Ronco C. 2023. The role of endotoxin in septic shock. Crit Care 27:400. doi:10.1186/s13054-023-04690-5 37858258 PMC10585761

[118] Li X, Li X, Huang P, Zhang F, Du JK, Kong Y, Shao Z, Wu X, Fan W, Tao H, Zhou C, Shao Y, Jin Y, Ye M, Chen Y, Deng J, Shao J, Yue J, Cheng X, Chinn YE. 2024. Acetylation of TIR domains in the TLR4-Mal-MyD88 complex regulates immune responses in sepsis. EMBO J 43:4954–4983. doi:10.1038/s44318-024-00237-8 39294473 PMC11535217

[119] Hofstaedter CE, O’Keefe IP, Met CM, Wu L, Vanderwoude J, Shin S, Diggle SP, Riquelme SA, Rasko DA, Doi Y, Harro JM, Kopp BT, Ernst RK. 2024. Pseudomonas aeruginosa lipid A structural variants induce altered immune responses. Am J Respir Cell Mol Biol 71:207–218. doi:10.1165/rcmb.2024-0059OC 38656811 PMC11299085

[120] Hofstaedter CE, Chandler CE, Met CM, Gillespie JJ, Harro JM, Goodlett DR, Rasko DA, Ernst RK. 2024. Divergent Pseudomonas aeruginosa LpxO enzymes perform site-specific lipid A 2-hydroxylation. MBio 15:e0282323. doi:10.1128/mbio.02823-23 38131669 PMC10865791

